# Effect of social rank in hair rams on the number of lambs sired and their postnatal development

**DOI:** 10.5194/aab-68-151-2025

**Published:** 2025-02-19

**Authors:** Estela Garza-Brenner, Fernando Sánchez-Dávila, Javier Hernández-Melendez, Keyla Mauleon-Tolentino, Rogelio Alejandro Ledezma-Torres, Marisol González-Delgado, Carlos Luna-Palomera, Cecilia C. Zapata-Campos, José Fernando Vazquez-Armijo

**Affiliations:** 1 Posgrado Conjunto, Facultad de Agronomía-Facultad de Medicina Veterinaria y Zootecnia, Universidad Autónoma de Nuevo León, General Escobedo N.L., General Escobedo, Nuevo León, CP 66050, Mexico; 2 Facultad de Ingeniería y Ciencias, Universidad Autónoma de Tamaulipas, Ciudad Victoria, Tamaulipas, CP 87274, Mexico; 3 División Académica de Ciencias Agropecuarias 86280, Universidad Juárez Autónoma de Juárez de Tabasco, Villahermosa, Tabasco, CP 86280, Mexico; 4 Facultad de Medicina Veterinaria y Zootecnia, Universidad Autónoma de Tamaulipas, Carretera Victoria-Mante km 5 s/n, Ciudad Victoria, Tamaulipas, CP 87274, Mexico; 5 Centro Universitario UAEM Temascaltepec, Universidad Autónoma del Estado de México, Temascaltepec, CP 51300, Mexico

## Abstract

The impact of social rank among hair rams on reproductive efficiency has been extensively studied, particularly regarding its influence on ewes and rams under various lambing scenarios, both within and outside the breeding season. However, limited information exists on the specific effects of social rank on lamb paternity. The present study aimed to evaluate the influence of social rank during a 35 d mating period on paternity outcomes and the postnatal development of lambs during the breeding season. A total of 108 adult ewes were divided into six groups, with 18 ewes per group. Each group was paired with two rams: one dominant ram (DRam) and one subordinate ram (SRam). Lamb development was monitored from birth through weaning and up to 150 d of age. Paternity was determined for 107 lambs using a panel of 116 single-nucleotide polymorphism (SNP) markers to assign sires from among the 12 rams included in the study. Social rank showed significant differences in lambing outcomes depending on the type of birth ( P<0.05 ). DRams sired 67 % of lambs, while SRams sired 33 %. DRams exhibited a significantly higher proportion of twin births (52.9 %) compared to SRams (32.4 %; P<0.01 ). However, no significant differences were observed between the ram groups for other lambing types. Lambing type also significantly influenced lamb weight gain, with single-born lambs achieving greater weight gain than twins and triplets ( P<0.05 ). Additionally, lamb sex ratios differed significantly between ram groups, with DRams producing a higher proportion of male lambs (50.7 %) compared to SRams (41.2 %; P<0.05 ). This study demonstrates that social rank significantly affects reproductive outcomes, including lamb paternity, lambing type, birth weight and body weight at 3 months of age. These findings highlight the importance of considering social hierarchy when managing breeding programs to optimize reproductive efficiency in hair sheep production systems.

## 1 Introduction

In ungulate mammals, key aspects of sexual behavior include the mechanisms regulating differential reproductive success among individuals (Sorin, 2004) and the competition that arises during mating within a group of females (Preston et al., 2005). Similarly, male competition for territory (in wildlife) or for achieving higher sexual status can provide a reproductive advantage (Sorin, 2004). In such scenarios, dominant males strive to maintain exclusive access to estrous females (Chapman et al., 2023). This behavior is particularly relevant in sheep, a promiscuous species, as ewes may mate with multiple males during their receptive period (estrus) (Preston et al., 2005; Chapman et al., 2023).

In sheep production systems, where the practice of using two or more rams in pens or extensive mating is common, social rank (SR) significantly influences mating behavior. Dominant rams tend to exhibit a stronger libido and display more effective sexual behaviors, such as mounting females in a shorter time (Aguirre et al., 2007).

However, the opposite can also be considered: when an ewe is in estrus and could choose a ram, she may be more attracted to the subordinate ram to avoid the aggressiveness of the dominant ram (Díaz et al., 2021). It has also been noted that both types of rams can show docility and boldness when covering a female in estrus, which can have a major impact on pregnancy rates, especially in adult rams of the bighorn species (*Ovis canadensis*) (De Young et al., 2006). However, in various regions of the world, rams aged 18 months and older may have problems covering ewes in estrus within sheep flocks. Up to one-third of these rams used for mating may be asexual, non-working rams with low sexual libido (Roselli et al., 2011). Specifically, regarding the effect of SR, a subordinate ram may show some of the limitations mentioned above, particularly in competitive scenarios. The most affected aspect is often the expression of low sexual libido, which significantly impacts the number of lambs born. By contrast, dominant rams with high sexual libido may sire 39 % to 70 % of the lambs born in a breeding season (Alexander et al., 2012). This suggests that when dominance is combined with good sexual performance, more ewes become pregnant (66 %) and more lambs are sired (68 %) than when sexual performance is low (34 % pregnant ewes and 32 % sired lambs) (Stellflug et al., 2006). These differences may be important depending on the scenario to which the high- and low-performing rams are exposed; i.e., when exposed to groups of ewes that are synchronized in estrus (high mating intensity), rams with high sexual performance mount more females and sire more lambs than rams with low sexual performance do (Stellflug et al., 2008). However, when natural mating occurs without pressure to cover ewes in estrus, there are no differences between the two groups of rams (Stellflug et al., 2008).

For this study, we assumed that hair rams will face a scenario of competition to cover an estrous ewe and that dominant rams will exhibit better sexual activity and therefore produce more offspring than subordinate rams (i.e., effect of social rank). Notably, few paternity studies involving hair rams, especially studies using molecular markers, have been performed to date. The objective of this study was to determine whether dominant rams affect ewe fertility and lamb weight gain compared to subordinate rams in an extensive flock.

## 2 Materials and methods

### 2.1 Study location

The fieldwork was performed in the Unidad Académica Marín of the Facultad de Agronomía of the Universidad Autónoma de Nuevo León, Marín, Nuevo León, Mexico, located at a latitude of 25°53^′^00^′′^ N and longitude of 100°02^′^00^′′^ W. The altitude at this location is 407 m, and it has a dry climate and a temperature ranging from 10 to 21 °C in winter and from 23 to 35 °C in summer. The present study was carried out in the winter season (January) with a breeding period of 35 d. Samples of blood were collected and sent to the Laboratorio Nacional de Nutrigenómica y Microbiómica Digestiva Animal (LANMDA) of Laboratorio de Biotecnología Animal (LBA) – Centro de Biotecnología Genómica of Instituto Politécnico Nacional (IPN), located in Reynosa, Tamaulipas, Mexico, for DNA extraction and processing for paternity tests.

### 2.2 Animal management

All ewes and rams used in this study were orally dewormed with 5 mg kg^−1^ Closantel (Grupo Lovet, Mexico City, Mexico) and received a single intramuscular dose of 500 000 IU vitamin A, 75 000 IU vitamin D and 50 mg vitamin E1 (Vigantol; Elanco, Mexico City, Mexico).

A total of 108 adult Saint Croix ewes, with a mean body weight of 33.2 ± 7.1 kg, an average age of 3.1 ± 0.2 years and a mean parity of 4.3 ± 0.4, were included in this study. The ewes were allocated into six groups of 18 individuals each based on body weight. Similarly, 12 adult rams of the same breed, with a mean live weight of 48 ± 5.7 kg, were paired into six dyads according to body weight.

#### Social rank of rams

The Synnott and Fulkerson (1984) feeding test was used to determine the social rank of the rams used in the study. This consisted of fasting the rams for 24 h and then isolating the pair in a pen away from the other rams where a single hole feeder was placed with food and only one of the two rams could access the food; the male that consumed food for 1 min was identified as the dominant and the other as the subordinate male.

### 2.3 Lamb management from birth to weaning

A total of 107 lambs born to 87 ewes were recorded during June and July, corresponding to the winter lambing period (January) of the same year. The lambs had a mean birth weight of 2.08 ± 0.71 kg and a mean weaning weight of 12.64 ± 2.79 kg. Of these, 53 were males and 54 females. The lambs were reared alongside their dams, which grazed daily for 7 h (08:00–15:00) in buffelgrass (*Cenchrus ciliaris* L.) paddocks. After grazing, the ewes were reunited with their offspring and remained together overnight, resuming grazing the following day.

During this time, the rams were kept in their pens during the day and joined the ewes in the evening after grazing. The lambs remained in the pens and were provided ad libitum access to a concentrate diet containing 18 % crude protein and 2.1 Mcal kg^−1^ of feed. They were weaned at 60 d of age and received appropriate healthcare, as previously described for the ewes and rams. Additionally, each lamb was intramuscularly administered 2.5 mL of a bacterin–toxoid (BOBACT 8; MSD Animal Health, Madison, NJ, USA) to prevent pneumonic pasteurellosis, symptomatic blackleg, malignant edema, gas gangrene, infectious necrotic hepatitis and enterotoxemia (pulpy kidney).

#### Lamb productivity variables

Lambs were weighed at birth; at weaning; and at 90, 120 and 150 d of age using a 300 kg crane scale (Walfront, Lewes, DE, USA). The sex of each lamb was recorded, as was the type of birth of each ewe and in which of the six groups each ram dyad was placed.

### 2.4 Paternity testing

In total, 12 rams from multiple winter matings were evaluated to determine the paternity of 107 lambs born during June and July. Blood samples were collected from each lamb at weaning and from each sire ram involved in the mating. The samples were obtained using 3 mL Monoject tubes (Cardinal Health, Dublin, OH, USA) containing tripotassium ethylenediaminetetraacetic acid (K3EDTA) as an anticoagulant and a 0.8×0.38 mm Vacutainer needle (Becton, Dickinson and Company, Franklin Lakes, NJ, USA).

After collection, samples were placed on ice and maintained at a temperature of 2 to 3 °C for immediate transport to the LANMDA-IPN laboratory. The transport time was approximately 3 h, ensuring prompt processing and DNA extraction for subsequent paternity analysis.

On receipt of the samples at the LANMDA-IPN, DNA extraction was performed using a commercial kit (GenElute Mammalian Genomic DNA Kit, Cat. G1N350; Sigma-Aldrich, St. Louis, MO, USA). The samples were then sent to the GeneSeek International Laboratory (Lincoln, NE, USA) for typing. The paternity of all samples (12 possible sire rams and 107 male and female progeny) was assigned using the GeneSeek Ovine-Global Sheep Parentage Panel, recommended by the International Society of Animal Genetics for the verification of paternity in sheep (Al-Atiyat et al., 2015; Tortereau et al., 2017). The panel of markers used in the study is shown in Table 1.

**Table 1:** Panel of 116 SNP-type markers for paternity testing of lambs born during the study.

CL635241	CZ920359	CZ920950	CZ925803	DU175804	DU182679	DU183112	DU183841
DU194639	DU202116v2	DU209581	DU213735	DU216457	DU223894	DU225323	DU231007
DU231335	DU232778	DU238011	DU245518	DU247686v2	DU258053	DU258149	DU260201v2
DU269694	DU271929v2	DU286106	DU295081	DU299578	DU300156	DU301502	DU301854v2
DU302760	DU310703	DU325267	DU325612	DU326572	DU328546	DU329154	DU348827
DU352764	DU362773	DU369175	DU380983	DU383209	DU383863	DU388282v2	DU396708
DU398082	DU405213v3	DU411403	DU413316	DU425376	DU426825	DU433863	DU440434v2
DU442796	DU446213	DU446965	DU452167	DU452456	DU453259	DU462008	DU462820
DU463532	DU464373	DU467751	DU471913	DU492723	DU511222	DU512685	DU519086
DU521806	DU522113	OAR10_68517121	OAR10_92199067	OAR11_56075682	OAR12_11657392	OAR14_19986506	OAR16_36737603
OAR16_64456388	OAR1_172310048	OAR1_227032731	OAR1_46249324	OAR21_14165572	OAR22_1023592	OAR22_40609932	OAR23_37250725v2
OAR24_17892863	OAR24_44850918	OAR25_34247335	OAR26_27421728	OAR26_6517460	OAR2_141253696	OAR3_145344922	OAR3_238210924
OAR5_110500655	OAR6_34448315	OAR6_88678679	OAR7_31647698	OAR8_30441759	OAR8_38564574	OAR8_57122732	OAR9_30296744
OAR9_46531990	OAR9_71172016	s03883	s13271	s17574	s19512	s37320	s39039
s51543	s64995	s73229	s75196				

The CERVUS 2.0 computer program (Marshall et al., 1998) was used for paternity assignment with multiple matings and exclusion probabilities in the presence or absence of parental genotypic information (Kalinowski ET AL., 2007); the genotyping error rate was 0.01, and the confidence was 80 % and 95 %. We evaluated the parent–child relationship of individuals according to the likelihood theory, where a positive value of limits of detection (LOD) indicated that the parent–child relationship was established; that is, the candidate father was the real parent. When there were two or more candidate fathers whose LOD value was greater than 0, the one with the higher LOD value was preferred. The program evaluates the feasibility of assigning paternity to the most likely sire by means of a simulation module in which delta criteria are set. The proportion of false positives in the assignments (confidence level) is defined according to the delta values obtained. Confidence levels can be set by the user from a relaxed (80 %) to a strict (95 %) level (Marshall et al., 1998; Slate et al., 2000).

### 2.5 Statistical analysis

A linear mixed-effects model with repeated measures over time was applied to estimate the effects of social rank and lambing type as independent variables on weights recorded from birth to 150 d of age. The sex of each offspring was included as a dependent variable. Interactions between the studied variables were analyzed but showed no significant differences and were therefore excluded from the final statistical model. The $\chi^{2}$ test was employed to determine the frequency and percentage of paternity assigned to each ram and to evaluate the influence of social rank (dominant vs. subordinate) through the offspring (lambs) sired by each ram. Differences between means for significant effects were identified using the least significant difference method, with statistical significance set at P<0.05 . All analyses were conducted using SPSS software (2013), version 22 (IBM Corp., Armonk, NY, USA).

## 3 Results

Table 2 presents the main effects analyzed in this study. A significant dominance effect was observed for lambing type ( P<0.05 ). Lambing type also significantly influenced birth weight; weaning weight; and body weights at 90, 120 and 150 d of age. Additionally, a notable difference in the sex ratio of lambs was identified between the two groups of rams, with a higher proportion of male lambs being produced by dominant rams (DRams) (50.7 %) compared to subordinate ram (SRams) (41.2 %) ( P<0.05 ).

**Table 2:** Main effects of evaluated factors: social rank, ram groups and lambing type in Saint Croix hair rams during the winter season in northeastern Mexico.

Source of	Lamb	Lambing	Birth	Weaning	Live weight	Live weight	Live weight
variation	sex	type	weight	weight (kg,	at 90 d	at 120 d	at 150 d
			(kg)	60 d)	(kg)	(kg)	(kg)
Social rank	0.02	0.05	ns	ns	ns	ns	ns
Lambing type	ns	–	0.009	0.001	0.004	0.05	0.02

Data are presented as P values. ns: not significant.

Table 3 summarizes the effects of social rank on lambing type in hair sheep lambs. Dominant rams (DRams) exhibited a significantly higher proportion of twin births (52.9 %) compared to subordinate rams (SRams), which had 32.4 % twin births ( P<0.01 ). No significant differences were observed for other lambing types ( P>0.05 ). Additionally, the sex ratio of the lambs did not differ significantly among the six groups of rams ( P>0.05 ).

**Table 3:** Effects of social rank, individually or in a group, on lambing type and sex of summer-born Saint Croix lambs.

	Lambing type			Lamb sex	
Ram code	Single	Twin	Triplet	Male	Female
1188D	11	13	0	10	14
5578D	0	1	0	1	0
5718D	2	7	3	5	6
574D	6	8	1	9	6
5763D	0	0	1	0	1
5768D	4	4	0	4	4
5777D	6	3	0	6	3
Total	28	36^a^	5	35	34
5686S	9	0	0	5	4
2211S	6	6	0	7	5
5766S	6	2	0	2	6
5780S	2	3	0	0	5
2084S	0	0	0	0	0
Total	23	11^*b*^	0	14	20

^a,b^ Data with different superscript letters within the same column are significantly different at P≤0.05 .

Table 4 shows the complete list of lambs born in summer whose mothers had mated in January. It shows the distribution of the lambs according to whether their sire was a DRam or an SRam.

**Table 4:** Distribution of rams by social rank in mating with hair sheep, with sire assignment based on microsatellite paternity testing.

Candidate ram sire				
Lamb ID	Dam ID	Ram sire	Ram sire	Assigned
		1	2	ram sire
07G	1569	2211S	5718D	1188D
105G	1840	2211S	5718D	2211S
106G	5673	2211S	5718D	2211S
107G	5705	2211S	5718D	2211S
11G	1569	2211S	5718D	5777D
120G	1567	2211S	5718D	2211S
132G	5705	2211S	5718D	5777D
134G	4232	2211S	5718D	5718D
20G	3222	2211S	5718D	5777D
31G	3634	2211S	5718D	2211S
40G	5695	2211S	5718D	5718D
47G	1687	2211S	5718D	2211S
52G	1564	2211S	5718D	2211S
54G	4031	2211S	5718D	5777D
66G	4202	2211S	5718D	5718D
69G	5746	2211S	5718D	2211S
72G	5745	2211S	5718D	2211S
81G	8F	2211S	5718D	2211S
93G	22F	2211S	5718D	5777D
99G	1567	2211S	5718D	2211S
02G	4177	574D	2084S	574D
04G	4178	574D	2084S	574D
101G	5694	574D	2084S	574D
108G	5757	574D	2084S	574D
112G	1540	574D	2084S	574D
35G	5712	574D	2084S	574D
37G	5712	574D	2084S	574D
43G	5759	574D	2084S	574D
45G	20F	574D	2084S	574D
71G	1545	574D	2084S	574D
80G	3030	574D	2084S	574D
89G	5749	574D	2084S	5686S
05G	5711	5766S	5768D	5768D
118G	1552	5766S	5768D	5768D
128G	5750	5766S	5768D	5686S
18G	5755	5766S	5768D	5768D
42G	16F	5766S	5768D	5766S
46G	573	5766S	5768D	5768D
57G	1539	5766S	5768D	5766S
59G	1557	5766S	5768D	5768D
64G	3031	5766S	5768D	5766S
74G	1563	5766S	5768D	5766S
76G	1539	5766S	5768D	5766S
77G	1542	5766S	5768D	5686S
78G	1568	5766S	5768D	5766S
79G	4F	5766S	5768D	5768D
97G	1552	5766S	5768D	5768D
100G	1317	5763D	5686S	1188D
102G	1317	5763D	5686S	1188D
103G	5753	5763D	5686S	5686S
110G	1555	5763D	5686S	5768D
116G	NL516	5763D	5686S	5686S

**Table 4:** Continued.

Candidate ram sire				
Lamb ID	Dam ID	Ram sire	Ram sire	Assigned
		1	2	ram sire
122G	569	5763D	5686S	5686S
124G	5697	5763D	5686S	5766S
19G	1561	5763D	5686S	574D
21G	1561	5763D	5686S	5718D
23G	1845	5763D	5686S	5686S
36G	5674	5763D	5686S	5686S
44G	560	5763D	5686S	1188D
67G	3032	5763D	5686S	5766S
01G	4128	5578S	1188D	1188D
03G	4127	5578S	1188D	1188D
06G	4179	5578S	1188D	1188D
09G	1546	5578S	1188D	1188D
126G	5752	5578S	1188D	1188D
12G	4180	5578S	1188D	1188D
130G	5754	5578S	1188D	1188D
14G	4181	5578S	1188D	1188D
15G	5748	5578S	1188D	1188D
25G	1550	5578S	1188D	1188D
33G	5717	5578S	1188D	5686S
39G	1558	5578S	1188D	1188D
41G	3033	5578S	1188D	1188D
50G	1558	5578S	1188D	2211S
55G	1688	5578S	1188D	1188D
58G	5692	5578S	1188D	1188D
60G	5692	5578S	1188D	574D
70G	1688	5578S	1188D	1188D
84G	4210	5578S	1188D	1188D
85G	572	5578S	1188D	1188D
86G	114A	5578S	1188D	1188D
88G	114A	5578S	1188D	1188D
96G	1548	5578S	1188D	1188D
98G	1566	5578S	1188D	5780S
104G	4218	5780S	5777D	5780S
13G	24F	5780S	5777D	5777D
17G	4134	5780S	5777D	5777D
24G	1554	5780S	5777D	574D
26G	1554	5780S	5777D	5718D
27G	4140	5780S	5777D	5777D
28G	1554	5780S	5777D	5718D
32G	1565	5780S	5777D	5763D
34G	1565	5780S	5777D	5718D
49G	4047	5780S	5777D	5718D
51G	4047	5780S	5777D	5718D
53G	5672	5780S	5777D	5777D
62G	3034	5780S	5777D	5780S
65G	5672	5780S	5777D	5718D
68G	4203	5780S	5777D	5780S
75G	1849	5780S	5777D	574D
87G	1544	5780S	5777D	5578S
90G	1686	5780S	5777D	5780S
95G	1544	5780S	5777D	5718D

ID, identification; D, DRams; S, SRams.

Table 5 also shows the weight gain of the lambs according to the type of birth, with single lambs showing significantly better weight gain than twin lambs and triplets ( P<0.05 ).

**Table 5:** Effect of ram group and lambing type on body development from birth to 150 d of age in summer-born Saint Croix lambs.

			Live weight (kg)		
Ram group	Birth weight (kg)	Weaning weight (kg, 60 d)	90 d	120 d	150 d
I	3.09 ± 0.15^a^	12.47 ± 0.62	16.40 ± 0.81^b^	19.42 ± 1.27	24.36 ± 1.15
II	2.92 ± 0.20^a^	11.55 ± 0.80	15.53 ± 1.04^b^	19.40 ± 1.60	22.41 ± 1.40
III	3.44 ± 0.18^a^	13.98 ± 0.72	19.15 ± 0.93^a^	23.32 ± 1.43	26.67 ± 1.30
IV	3.27 ± 0.19^a^	12.35 ± 0.77	16.09 ± 1.00^b^	19.88 ± 1.54	24.39 ± 1.35
V	3.22 ± 0.14^a^	12.94 ± 0.57	17.89 ± 0.74^b^	20.94 ± 1.13	26.07 ± 1.04
VI	2.59 ± 0.16^b^	12.28 ± 0.64	16.86 ± 0.83^b^	19.83 ± 1.27	25.16 ± 1.15
Lambing type					
Single	3.27 ± 0.09^a^	13.69 ± 0.36^a^	18.21 ± 0.49^a^	21.72 ± 0.76^a^	26.21 ± 0.67^a^
Twin	2.93 ± 0.10^a^	11.57 ± 0.38^b^	15.83 ± 0.52^b^	19.05 ± 0.81^b^	23.59 ± 0.74^b^
Triplet	2.47 ± 0.31^b^	11.19 ± 1.16^b^	16.05 ± 1.57^b^	19.22 ± 2.42^b^	22.97 ± 2.11^b^

^a,b^ Data with different superscript letters within the same column are significantly different at P≤0.05 .

## 4 Discussion

Consistent with the hypothesis of our study, social rank was found to have a greater influence on the number of lambs sired by dominant rams (DRams) compared to subordinate rams (SRams) across all rams evaluated. DRams sired 71 lambs (66.4 %), whereas SRams sired 36 lambs (33.6 %).

Similar results have been reported in previous studies (Stellflug et al., 2006), where DRams and SRams sired 68 % and 32 % of the offspring, respectively. However, a key difference in our study was the use of natural mating conditions with ewes, whereas the study by Stellflug et al. (2006) utilized estrus synchronization in White-faced ewes. According to preliminary studies and personal communication with Fernando Sánchez-Dávila (2024), no differences in sexual behavior were observed between the two groups of rams.

Although not confirmed, it is hypothesized that the higher reproductive success of DRams might be attributed to superior semen quality. However, a recent study by Mauleón et al. (2023) reported no differences in semen quality among hair sheep rams at the same latitude as the present study. This suggests that other factors, such as sexual libido, may explain the reproductive performance of SRams. For instance, SRams 5766S, 5686S and 2211S sired 8, 9 and 12 lambs, respectively, surpassing their DRam counterparts within the same groups.

In fact, of the 12 rams evaluated, only 1 sired no lambs (2084); this ram was in group II. Furthermore, all rams were bred together until adulthood, including the study period. This is consistent with the study protocols of Stellflug et al. (2006) and Katz (2008), in which up to 8 % of rams were male-oriented when bred together; they were not interested in ewes in estrus. This is relevant in our study because it involved hair rams adapted to the semi-desert regions of northeastern Mexico, which show the same pattern of sexual behavior previously reported by Sánchez-Dávila et al. (2020), i.e., that sexual behavior may be more strongly influenced by the time of mating and, in young lambs, by the time of lambing (Sánchez-Dávila et al., 2019).

In our study, the mating season lasted 35 d, allowing sufficient time for the rams to mate with the ewes twice. This strategy resulted in high pregnancy and lambing rates across the ram groups. Similarly, Stellflug et al. (2006) reported that short 21 d mating periods yielded low percentages of ewes requiring a second service ( <3% ).

Paternal genotyping data in our study revealed that among eight sets of twin lambs, both DRams and SRams sired one lamb each. In one set of triplets, two lambs were sired by a DRam and one by an SRam. These findings suggest that individual rams can sire a greater number of lambs regardless of their social rank, indicating possible differences in reproductive performance among the groups evaluated.

Juengel et al. (2019) previously reported that ram age significantly influences mating success, with adult rams ( >3 years) achieving a 15 %–20 % higher success rate compared to younger rams (1–2 years). In our study, the use of adult rams ( >3 years) combined with an appropriately timed mating season contributed to the high reproductive success observed in the hair sheep rams. According to Juengel et al. (2019), this phenomenon may be repeatable (40 %) and heritable (26 %).

Moreover, Bench et al. (2001) reported that 82.0 % of lambs with high reproductive performance were sired by rams exhibiting high sexual behavior, whereas 59.5 % of lambs with low reproductive performance were sired by rams with low sexual behavior. These findings emphasize the importance of selecting rams with favorable reproductive traits to optimize lambing outcomes.

Regarding the paternity analysis, we found that, among the 12 rams evaluated, only one had no paternity assigned to any offspring within its designated group. With regard to the paternity analysis carried out by the National Laboratory of Animal Nutrigenomics and Digestive Microbiomics (LANMDA) of the Animal Biotechnology Laboratory of the Genomic Biotechnology Center of the IPN, we found that, after typing the animals with the 116 SNP panels recommended by the ISAG for the verification of paternity in sheep, of the assignments among the 12 rams evaluated, only one did not have paternity assigned to any offspring within its group. However, according to the laboratory's calculations, the assignments have a reliability of 99.99 %. In the results report, they mentioned that when they found that none of the sires assigned to the pen turned out to be the biological father, they opted for a reassignment analysis using all the sires sent from the list and were able to assign the paternity of the missing offspring with 99.99 % confidence. Although the study was carried out with control and management of the facilities, these were not sufficient to attribute this result to the dominance of the identified biological sire, who could have entered the pen of this group of females due to a higher reproductive drive (Kabasakal, 2023). It is important to mention the advantage of conducting a paternity test because despite the controlled mating during the study, the rams migrated out of the pen, which is a common occurrence in herds, thus designating a false paternity and therefore attributing erroneous racial and production characteristics to the offspring by not carrying out DNA tests.

Calus et al. (2019) similarly reported consistent parentage assignment results, even when offspring and potential parent lists were excluded from the analysis. Their study confirmed that parentage assignment reliably identifies the correct parent–offspring pairs and distinguishes between parents and offspring, irrespective of the inclusion of a predefined list of possible progenitors.

Another possible explanation could involve biological factors, such as kinship with the excluded ram. For instance, paternity assignment may be influenced by genetic similarity, especially in cases of homozygous twins (Cunningham et al., 2022), although this was not confirmed in our study. Furthermore, epigenetic factors have been reported to influence cellular conformation and gene expression, potentially altering phenotypic traits even among homozygous twins (Arista, 2019) and inbreeding populations (Marsh et al., 2017).

All rams in the study were tested and evaluated for reproductive performance, ruling out infertility due to poor semen quality. Semen collection and evaluation were conducted at the start of the study to assess fertility and ensure comparability between ram groups. Parameters assessed included ejaculate appearance (volume and color) and general sperm morphology, viability, concentration and motility.

Paternity and parentage tests based on SNP panels are widely validated and employed in several countries and species of zootechnical interest, including sheep (Souza et al., 2012; Sheriff and Alemayehu, 2018). These tests enable reliable paternity assignment, even in scenarios involving multiple potential sires (Laughlin, 2001; Domínguez-Viveros et al., 2020).

In terms of lambing type, more offspring from twin births were sired by DRams than SRams. In flocks with multiple matings this difference can be improved in the long term by selecting DRams with high fertility. Despite the low heritability values (0.046–0.100), this offers an opportunity to improve income by increasing the number of offspring per reproductive cycle (Schmidová et al., 2016a, b). According to De Lima et al. (2020), fertility deserves special attention in selection programs because it can lead to higher profitability. In this case, fertility is defined as the number of ewes lambing over the number of ewes that mated, and fecundity refers to the average number of offspring born per female. High fertility has a positive effect on the total number of animals marketed and the replacement rate. In general, small ruminants have a higher frequency of multiple lambings, and the average litter size of ewes reportedly varies from 1.3 to 2.3 (Sánchez-Dávila et al., 2015).

In our study, the only differences in weight gain found between the groups of rams evaluated were in birth weight and weight at 90 d of age. This suggests that lambs sired by DRams had better weight gain, possibly because the groups with a higher number of single births contributed more to pre- and postnatal development compared to those with multiple births (López-Carlos et al., 2021). Notably, another study showed that when ewes were supplemented according to their nutritional needs during pregnancy, birth weight and postnatal development were not affected (Tygesen et al., 2008). A study also confirmed that single-birth lambs showed better physical development from birth to 150 d of age than twin- and triple-birth lambs (Monforte et al., 2024).

## 5 Conclusions

In summary, our results showed that under controlled mating during the breeding season, DRams sired a higher percentage of lambs than SRams did. However, SRams develop opportunistic strategies, allowing them to sire up to 32 % of lambs depending on their mating ability. Furthermore, lambs sired by DRams had higher birth weights and reached higher weights at 90 d of age. These results highlight a new possibility of establishing mating strategies between hair rams of different social ranks to obtain higher numbers of lambs, regardless of whether the parents are dominant or subordinate, and to influence the productivity of the flock.

## Data Availability

The original data are available upon request to the corresponding author.
